# High-quality AlN grown with a single substrate temperature below 1200 °C

**DOI:** 10.1038/s41598-017-07616-8

**Published:** 2017-08-02

**Authors:** Chun-Pin Huang, Kapil Gupta, Chao-Hung Wang, Chuan-Pu Liu, Kun-Yu Lai

**Affiliations:** 1 Department of Optics and Photonics, National Central University, Chung-Li, 320 Taiwan; 2 Department of Materials Science and Engineering, National Cheng Kung University, Tainan, 701 Taiwan

## Abstract

1.5-μm AlN grown by metal-organic chemical vapor deposition (MOCVD), with a single substrate temperature of 1180 °C, exhibits atomically flat surface and the XRD (102) peak width of 427 arcsec. The results are achieved with a pulsed NH_3_-flow condition, serving as an alternative for the commonly used temperature-varied buffer structure, which is often complicated and time-consuming. Inserting two pulsed-NH_3_-flow AlN layers in the epitaxial structure not only releases the lattice strain *via* the formation of three-dimensional nano-islands, but also smoothens the surface with prolonged lateral migration of Al adatoms. This effective growth technique substantially simplifies the manufacture of device-quality AlN.

## Introduction

Ever since the demonstration of smooth AlN by Ohba *et al*. in 1996^1^, the growth of high-quality AlN has been intensively pursued by numerous groups. These research efforts are mainly driven by the wide bandgap energy (6.2 eV) and exceptional piezoelectric properties of AlN^2,3^, making the binary an essential material for ultraviolet emitters and ultrasensitive detectors^4,5^. Despite the significant progress over the decades, attaining device-quality AlN still remains challenging.

One of the hurdles is the high growth temperature. Compared with the case of GaN, the much larger cohesive energy of AlN necessitates higher synthesis temperature to promote chemical reaction and atomic migration on the substrate surface^6^. For most commercial epitaxial systems, the high growth temperature (>1200 °C) of AlN requires specially designed reactor, which substantially increases the manufacturing cost^7^. Moreover, in order to alleviate the huge lattice strain between AlN and the sapphire substrate, it is often indispensable to insert low-temperature (LT, < 1000 °C) buffer layers under the high-temperature AlN^8–10^. Because of the difficulty in rapid and precise control over the RF-heater (or heating filament), changing the substrate temperature can be a very time-consuming process, particularly for the buffer growth involving periodic temperature oscillation to mitigate the undesired strain^8,9^. The high temperature and long growth time of AlN are among the keys reasons for the pricey ultraviolet light emitting diodes.

In this study, we demonstrate a growth tactic to achieve LT-buffer-free yet high-quality AlN with a single substrate temperature of 1180 °C, which is scarcely reported to date. The high-quality AlN is directly deposited on sapphire substrate by MOCVD, employing two morphology-engineering layers attained with pulsed NH_3_ supply. The pulsed-flow growth condition has been demonstrated as an effective route to enhanced lateral migration of the constituent atoms, hence the smoothened epitaxial surface^11–15^. Here we optimize the pulsed duration of NH_3_ flow and achieve a 1.5-μm AlN with the full width at half maximum (FWHM) of 427 arcsec determined from the x-ray diffraction (XRD) (102) peak. The observation with atomic force microscopy (AFM) and transmission electron microscopy (TEM) shows that the pulsed NH_3_ flow not merely renders atomically flat surface, but also annihilates the rampant threading dislocations by bending/merging them through the formation of three-dimensional (3D) nanoislands. These results hold great promise for the commercialization of AlN-based devices.

## Results

**Figure 1:**
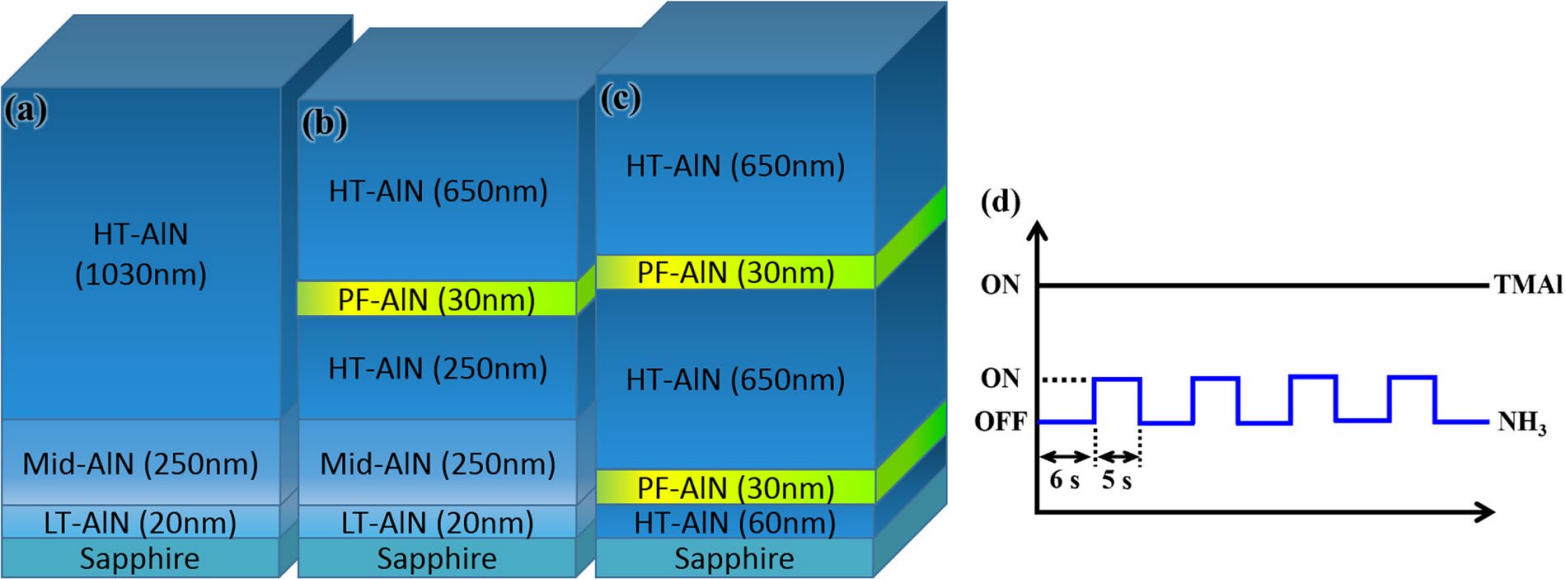
Layer structures of the (**a**) conventional CF sample; (**b**) SPF sample; (**c**) DPF sample. (**d**) Schematic illustration of the valve operation during the PF-AlN growth in (**b**) and (**c**). The on/off duration of NH3 flow is optimized to attain the atomically flat surface.

Figure 1(a–c) show the three studied AlN layer structures: Fig. 1(a) displays the AlN layers grown with conventional continuous flow (CF) conditions, hereafter referred as the CF sample; Fig. 1(b) and (c) present the layers achieved with single-pulsed-flow (SPF) and double-pulsed-flow (DPF) NH_3_ precursors, referred as the SPF and the DPF sample, respectively. Schematic illustration of the pulsed-flow valve operation is shown in Fig. 1(d). For the CF sample, the in-plain epitaxial strain is mitigated by the layers of low-temperature AlN (LT-AlN) and middle-temperature AlN (Mid-AlN), which were grown at 950 °C and 1030 °C, respectively. Since the growth temperature below 1000 °C favors 3D island formation, the LT-AlN was employed to produce the nucleation centers for subsequent crystal coalescence^10^. The Mid-AlN was adopted to alleviate lattice strain and to bend the dislocations as the enlarged AlN islands coalesced at the increased temperature^8,9^. The temperature is further increased to 1180 °C for the high-temperature AlN (HT-AlN) to promote lateral diffusion length^16^. To evaluate the effectiveness of pulsed-flow NH_3_ on crystal qualities, a thin (~30 nm) layer of pulsed-flow AlN (PF-AlN) attained at 1180 °C was introduced in the HT-AlN, as designated in Fig. 1(b). For the DPF sample in Fig. 1(c), the LT-AlN and Mid-AlN layers were replaced with a second PF-AlN inserted in HT-AlN. Growth parameters of the two PF-AlN layers in the DPF sample are identical. The growth rates of PF-AlN and HT-AlN are respectively around 0.183 nm/cycle, 4.29 nm/min, which can be raised by an increased TMAl flow.

It has been shown that the PF condition of gas flows in MOCVD growth can meritoriously improve the lattice structure of III-nitrides with reduced defect densities and smoothened surface^11–15^. The improvement was due to the strain release *via* the formation of 3D nano-islands during the interruption of reactants^12^. After the nucleation of the 3D islands, the interrupted source supply also allows the defective surface to be partially etched by the H ions and promotes lateral atomic migration on the surface so that the occurrence of pits and abnormal epi-structures can be prevented. The PF growth of AlN can be carried out with various off/on combinations of TMAl and NH_3_ supplies, i.e. either or both of the precursor flows can be paused with prudently controlled durations. Here, we adopt a simple combination by merely interrupting the NH_3_ flow with the off/on = 6-sec/5-sec, while keeping the TMAl flow continuous. The off duration of NH_3_ flow must be selected in consideration of the detrimental effect brought by the metal-rich condition. While properly prolonged NH_3_ interruption benefits epitaxial growth, excessive interrupted NH_3_ flow could lead to undesired accumulation of Al atoms, which instead degrades the crystallization process.

**Figure 2:**
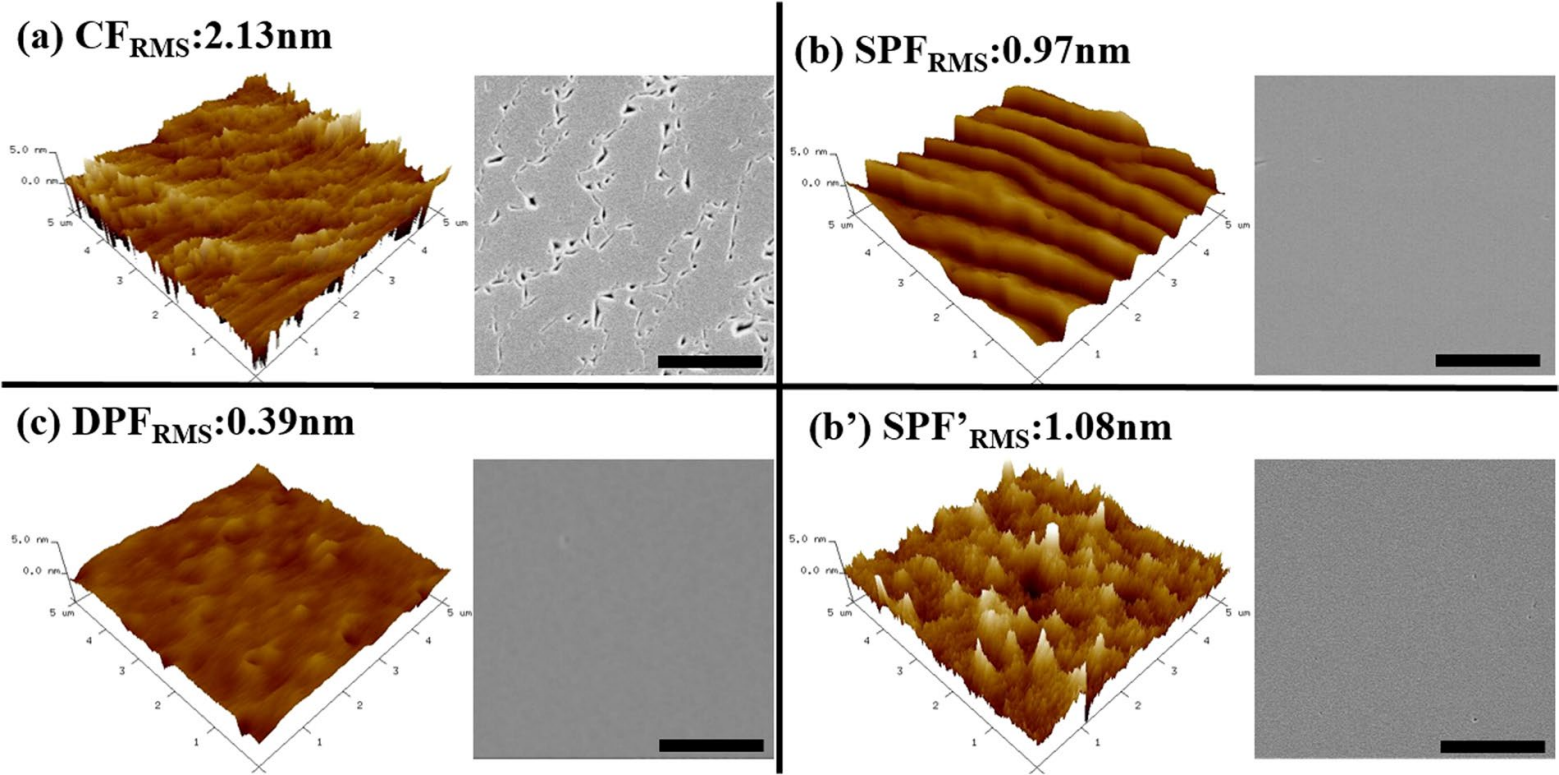
AFM (5 × 5 μm^2^) images (on the left) with corresponding RMS roughness and SEM images (on the right) recorded on the (**a**) CF sample; (**b**) SPF sample; (**b’**) SPF sample with the growth stopped at the red dash line indicated in Fig. 1(b); (**c**) DPF sample. Scale bars in the SEM images are 1 μm. It can be seen that the surface smoothness is effectively improved by the PF condition.

Figure 2 shows AFM and scanning electron microcopy (SEM) images recorded on the surface of the three samples. In order to study the role of the PF-AlN layer, Fig. 2(b’) presents the images taken upon the completion of PF-AlN, i.e. the surface indicated by the red dash line in Fig. 1(b). In Fig. 2(a), one can see the root-mean-square (RMS) roughness is 2.13 nm, being the highest among the measured results. The highest RMS roughness on the CF sample is due to the pits densely populated on the surface, which are less apparent in AFM owing to the deep holes below the surface but become evident in the associated SEM image. As the growth temperature (1180 °C) of HT-AlN is lower than most of those used for high-quality AlN^15,17–20^, it is believed that the dense pits are caused by insufficient atomic migration on the reactive surface. In other words, since the Al adatoms fail to reach the energetically favorable nucleation sites, the growth mechanism in HT-AlN of the CF sample is not fully converted from 3D mode (initiated in LT-AlN) to 2D (two-dimensional) mode^16^, which is the favored layer-by-layer process.

Evidenced by Fig. 2(b), the pit issue is solved on the SPF sample attained by interrupting the NH_3_ supply during the HT-AlN growth. Unlike the case in the CF sample, where the synthesis of AlN immediately takes place once the TMAl:NH_3_ adduct or the monomethyl-Al (MMAl, decomposed from TMAl) reach the reactive surface^21,22^, the Al precursors decomposed during the NH_3_ pulse interval are more likely to diffuse to the vacant atomic sites (created either by inherent in-plane strain or by H_2_ partial etching)^23^, and thereby flatten the pitted surface. The other benefit of pulsed NH_3_ flow can be explained using Fig. 2(b’), where a 3D hillocky morphology is seen on the immediate surface of PF-AlN. Albeit with the 3D texture, the RMS value in Fig. 2(b’) is found to be lower than that in Fig. 2(a). Since the 3D growth is generally regarded as an effective route to relieve the undesired lattice strain, the nanoscale hillocks seen in Fig. 2(b’) should facilitate the nucleation process in HT-AlN. Summarily, the PF condition not only prolongs the diffusion length of Al adatoms, but also ease the build-up of lattice strain by redistributing the atomic arrangement, both of which contribute to the elimination of defective surface pits. More importantly, when the layers of LT-AlN and Mid-AlN are replaced with another PF-AlN, the surface quality is improved even further, i.e. RMS roughness is decreased from 0.97 nm in Fig. 2(b) to 0.39 nm in Fig. 2(c), where the 2D atomic terraces become finer and denser. The result indicates that the nano-hillocks introduced by the PF growth can serve as the initial nucleation centers to accommodate the huge strain between AlN and sapphire, and the residual strain can be further mitigated by the second PF layer.

The improved surface quality by PF-AlN is also confirmed by XRD characterization, with which the full-width at half-maximum (FWHM) of the peaks in rocking curves (ω-scans) are summarized in Table 1. Since broadening of the symmetric reflection is subject to the prevalence of screw and mixed dislocation while the asymmetric one is hinged on edge dislocation^24^, the densities of dislocation in the three samples can be estimated by FWHMs of the (002) peak (symmetric) and the (102) peak (asymmetric), using the relationship:^25–29^

1 $$N=\frac{FWH{M}^{2}}{4.35|b{|}^{2}}$$

where N is the dislocation density, and b is the Burgers vector.

**Table 1:** Crystal properties of the three AlN samples. N_S+M_ is the calculated density of screw and mixed dislocation; N_E_ is the one of edge dislocation.

Sample	AFM RMS (nm)	FWHM of XRD(002) (arcsec)	FWHM of XRD(102) (arcsec)	N_S+M_ (cm^−2^)	N_E_ (cm^−2^)
CF	2.13	210	573	9.6 × 10^7^	3.7 × 10^9^
SPF	0.97	194	554	8.2 × 10^7^	3.4 × 10^9^
DPF	0.39	219	427	1.1 × 10^8^	1.9 × 10^9^

The calculated values of N are included in Table 1, where one can see that the densities of screw dislocations are similar among the three samples. The result is due to the fact that the strain energy arising from the lattice mismatch between AlN and c-plane sapphire is mostly released through the formation of edge dislocations, whose Burgers vectors are along the film/substrate interface, and thus leaves the symmetric (002) planar spacing essentially undistorted, i.e. the FWHM of (002) peak is insensitive to edge dislocations^18^. As the density of edge dislocations is mostly reflected by the width of the asymmetric (102) peak, the effect of PF-AlN is manifested by the decreased NE (edge dislocation density) in Table 1. For the DPF sample, with its NE being 49% less than that of the CF sample, the XRD FWHMs and dislocation densities are comparable to the high-quality AlN attained with temperature-varied buffer structures^15,17,30^. In Figures S1 in Supplementary Information, it is shown that the (102) peak width can be further reduced to 378 arcsec for a 3-μm-thick AlN with DPF-NH_3_ condition.

**Figure 3:**
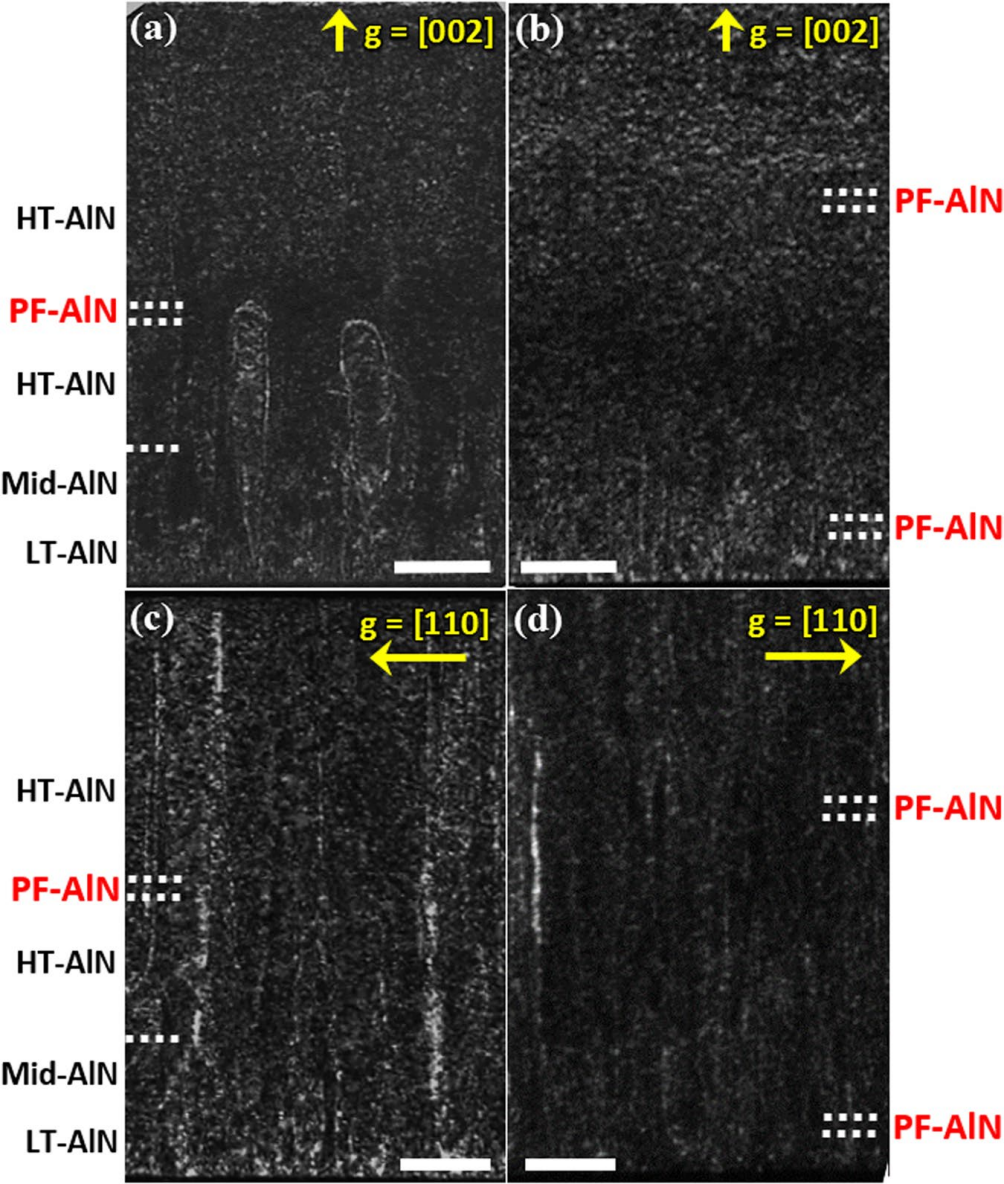
Cross-sectional dark-field TEM images of the AlN layers taken under two beam conditions. g = [002]: (**a**) the SPF sample and (**b**) the DPF sample. g = [110]: (**c**) the SPF sample and (**d**) the DPF sample. Dislocation bending is clearly seen in (**a**). Scale bars are 200 nm.

The role of PF-AlN on the propagation of threading dislocations can be visually revealed by cross-sectional TEM images taken with the SPF and the DPF samples, presented in Fig. 3. Because of the similar reasons in XRD characterization, the images of screw and edge dislocations are obtained using the diffraction vectors of g = [002] and g = [110], respectively^31^. The approximate positions of PF-AlN and other layers indicated in the images are determined by growth time and rates, which were calibrated with several experimental runs at prolonged growth time. In Fig. 3(a) and (b), it is seen that the dislocations (visualized as white threads by the weak-beam dark-field condition) sprout from the AlN/sapphire interface and propagate toward the (002) surface. Noted that, in the bottom region, the population of dislocations is less dense in Fig. 3(a) than in Fig. 3(b), suggesting that the LT-AlN is a superior nucleation layer to HT-AlN in terms of the capability to prevent the formation of screw dislocations. In Fig. 3(a), it is clear that the dislocations bend laterally in the region around the PF-AlN layer, and merge with other dislocations, forming half loops and thus curbing the upward spreading. Similar results were reported by other groups^8,9^. The effectiveness of PF-AlN is also viewed in Fig. 3(b), as one can see that the dislocation density becomes much less in the area above the first PF-AlN. The observation is consistent with previous works showing that most screw dislocations bend from the [002] direction within the first 0.5 μm^32^. The difference in dislocation density is further manifested by comparing Fig. 3(c) and (d), where the prevalent edge dislocations are found to be considerably reduced in the DPF sample. As threading dislocations are generally terminated with pits on epitaxial surface^33^, the much less dislocation densities of the DPF sample should contribute to the disappearance of surface pits discussed in Fig. 2. These TEM findings, echoing with the XRD results, unambiguously demonstrate that the double PF growth scheme can effectively improve the crystal qualities of AlN by suppressing the propagation of threading dislocations.

## Conclusion

High quality AlN with the XRD (102) peak width of 427 arcsec was demonstrated by MOCVD at a single substrate temperature of 1180 °C. The LT-buffer-free and atomically smoonth epitaxy was achieved by two separated AlN layers grown with pulsed NH_3_-flow condition. The pulsed NH_3_ supply facilitated crystal nucleation through the formation of 3D nano-islands, and flattened the surface *via* the prolonged atomic migration. The partial H_2_ etching during NH_3_ interruption also improved surface quality by removing the defective sites. Replacing the time-consuming LT-buffer with the PF-AlN layers should effectively simplify the growth procedure for device quality AlN, benefiting the production of deep ultraviolet emitters, sensors, and transistors.

## Methods

### MOCVD process

The AlN layers were grown on c-plane sapphire substrates by MOCVD (AIXTRON 200/4 RF), employing trimethylaluminum (TMAl) and ammonia (NH_3_) as the precursors for Al and N, respectively. H_2_ was used as the carrier gas. Growth pressure is 25 mbar. V/III ratio is 5623 for Mid- and LT-AlN, and decreased to 103 for HT-AlN.

### Measurement

Surface roughness of the epitaxial surface was measured by AFM in tapping mode. SEM images were recorded with field emission HITACHI S-4300 at the acceleration voltage of 10 kV. TEM images were taken with a JEOL 2100 F system at 200 kV, and the samples were prepared by focus ion beam using Ga ions at 30 kV.

## Electronic supplementary material

Supplementary Information
