# Author Correction: High-quality AlN grown with a single substrate temperature below 1200 °C

**DOI:** 10.1038/s41598-018-25248-4

**Published:** 2018-05-08

**Authors:** Chun-Pin Huang, Kapil Gupta, Chao-Hung Wang, Chuan-Pu Liu, Kun-Yu Lai

**Affiliations:** 1 Department of Optics and Photonics, National Central University, Chung-Li, 320 Taiwan; 2 Department of Materials Science and Engineering, National Cheng Kung University, Tainan, 701 Taiwan

Correction to: *Scientific Reports* 10.1038/s41598-017-07616-8, published online 02 August 2017

Kapil Gupta was omitted from the author list in the original version of this Article. This has been corrected in the PDF and HTML versions of the Article, and in the accompanying Supplementary Information file.

The Author Contributions section now reads:

C.P.H. ran the MOCVD growth, performed AFM, SEM and XRD measurements, and wrote the paper. K.G., C.H.W. and C.P.L. carried out the TEM characterization. K.Y.L. directed the research and wrote the paper. All authors reviewed the manuscript.

