# Supplementary material for: High-quality AlN grown with a single substrate temperature below 1200 °C
Source: Sci Rep. 2017 Aug 2;7:7135. doi: 10.1038/s41598-017-07616-8 (PMC5541044; doi:10.1038/s41598-017-07616-8)
Supplement: Supplementary file 1 — Supplementary Information [file 41598_2017_7616_MOESM1_ESM.doc]

**High-quality AlN grown with a single substrate temperature below 1200 ºC**

**Supplementary Information**

*Chun-Pin Huang^a^, Kapil Gupta^b^, Chao-Hung Wang^b^, Chuan-Pu Liu^b^, and Kun-Yu Lai^a,*^*

^a^ Department of Optics and Photonics, National Central University, Chung-Li 320, Taiwan.

^b^ Department of Materials Science and Engineering, National Cheng Kung University, Tainan 701, Taiwan.R.O.C.

*^*^* Authors to whom any correspondence should be addressed.

E-mail: kylai@ncu.edu.tw

Phone: +886-3-4227151 ext 25256


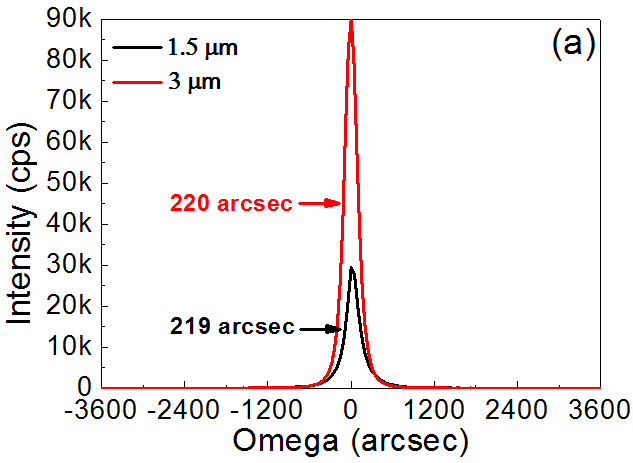

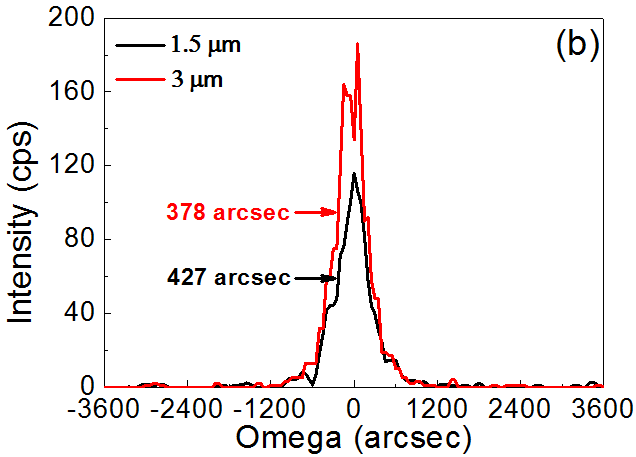


**Figure S1.** XRD scans of the AlN layers grown by MOCVD with the pulsed-flow NH_3_ condition. Rocking curves of the (a) (002) and (b) (102) diffractions, recorded on the AlN with the layer thicknesses of 1.5-μm and 3-μm. For the 3-μm AlN, the FWHM is reduced to 378 arcsec, rendering edge dislocation density of 1.4×10^9^ cm^-2^, according to Eq. (1) in the main text.
